# Advanced Endometrial Cancer Mimicking Pelvic Inflammatory Disease in a Postmenopausal Woman—A Case Report

**DOI:** 10.3390/life16101681

**Published:** 2026-10-08

**Authors:** Bogdan Mihnea Ciuntu, Irina Mihaela Abdulan, Lili Gabriela Lozneanu, Adelina Tanevski, Cosmin Velnic, Dana Petronela Romasc, Mihai Andronic, Andrei Chicos, Roxana-Elena Ciuntu, Dan Constantin Andronic

**Affiliations:** 1 Faculty of Medicine, “Grigore T. Popa” University of Medicine and Pharmacy, 700115 Iasi, Romania; bogdan-mihnea.ciuntu@umfiasi.ro (B.M.C.); lili_lozneanu@yahoo.com (L.G.L.); papancea.adelina@yahoo.com (A.T.); cosmin.velnic@gmail.com (C.V.); romasc.petronela@umfiasi.ro (D.P.R.); mdcandronic@gmail.com (M.A.); chicosandrei1@yahoo.com (A.C.); roxana-elena.ciuntu@umfiasi.ro (R.-E.C.); candronic@gmail.com (D.C.A.); 2 Department of General Surgery, County Clinical Emergency Hospital St. Spiridon, 700111 Iasi, Romania; 3 Ophthalmology, 2nd Surgery Department, Saint Spiridon Emergency Hospital, 700111 Iasi, Romania

**Keywords:** endometrioid endometrial carcinoma, acute salpingitis, recurrent post-menopausal malignancy, FIGO staging, surgical oncology

## Abstract

**Background:** Endometrial cancer is the most common gynecological malignancy in high-income countries, with recurrence occurring in approximately 8.3% of endometrioid endometrial carcinoma cases. Recurrence in elderly patients is associated with significantly worse outcomes, compounded by comorbidity burden, atypical clinical presentations, and the technical demands of salvage surgery. The co-occurrence of recurrent endometrioid endometrial carcinoma and acute salpingitis in an elderly patient represents a clinically complex and poorly characterized entity, with no systematic documentation in the current literature. **Case Presentation:** We report the case of a 78-year-old female patient who underwent open supracervical hysterectomy with bilateral adnexectomy for a large pelvic mass. Histopathological analysis confirmed locally advanced infiltrative endometrioid endometrial carcinoma, FIGO stage IIIA (pT3aNxMx), with concurrent acute purulent salpingitis and parasalpingeal abscesses of the left adnexa. An aggressive tumor progression of residual disease was identified at five months postoperatively, with CT imaging revealing a persistent uterine mass in direct contact with the bladder and a congenitally duplicated left ureter. Following multidisciplinary preoperative planning and Double-J catheter insertion, salvage completion surgery was successfully performed, including radical excision of the cervical stump and concurrent surgical reconstruction of the involved duplicated left ureters via direct bladder reimplantation and end-to-end anastomosis. The second histopathological examination confirmed recurrent locally advanced endometrioid endometrial carcinoma. **Conclusions:** Incomplete index resection with involved margins in advanced-stage (FIGO IIIA) high-risk endometrioid endometrial carcinoma can permit persistent residual tumor to progress aggressively. Salvage completion surgery in elderly patients with concurrent comorbidities and anatomical variants is highly complex and should be centralized in specialized units with integrated oncological, urological, and perioperative expertise.

## 1. Introduction

Endometrial cancer (EC) is the most common gynecological malignancy in high-income countries and the sixth most diagnosed cancer in women worldwide, with an estimated 417,367 new cases and 97,370 deaths recorded in 2020 [1]. Global incidence has been rising consistently over the past three decades; from 1990 to 2021, incidence rates among postmenopausal women aged 55 years and above increased progressively, with projections indicating a further 6.5% rise by 2036, driven primarily by increasing rates of obesity, type 2 diabetes mellitus, and hypertension [2]. In all countries studied, incidence rates in women aged 50 years and older are around 15 times higher than in younger cohorts, firmly establishing EC as a disease predominantly of postmenopausal and elderly women [3,4].

Endometrioid endometrial carcinoma (EEC) accounts for approximately 80% of EC cases and is classified as an estrogen-dependent tumor with a favorable prognosis at early stages [5]. Established risk factors for recurrence include high histological grade (G3), myometrial invasion of ≥50%, substantial lymphovascular space invasion (LVSI), advanced FIGO stage, lymph node metastasis, and tumor diameter exceeding 2 cm [5,6]. Outcomes for advanced and recurrent disease remain poor, with 5-year overall survival rates of 20–25% [5]. In population-based cohorts, recurrence occurs in approximately 8.3% of patients with EEC, with a median time to recurrence of 22.5 months; isolated vaginal recurrence, representing 27.2% of all recurrent cases, carries a significantly more favorable prognosis than pelvic, peritoneal, or distant disease [7]. High-grade EEC has a recurrence rate of approximately 16% [8].

Age is an independent adverse prognostic factor in EC. Older patients have significantly lower five-year event-free, overall, and cause-specific survival, and their recurrence rates are significantly higher than those of younger patients, independent of histological grade, FIGO stage, and treatment modality [9]. Elderly women are more likely to be undertreated because of comorbidity burden and diminished performance status, and the rate of treatment omissions increases proportionally with age, up to 27% [10]. Among women aged ≥70 years, non-endometrioid histological subtypes are significantly more prevalent, with serous carcinoma accounting for 20% of tumors in this age group compared with 3% in women under 50 years of age [11].

The surgical management of recurrent EC requires individualized, multidisciplinary evaluation at specialized centers, guided by the site and extent of recurrence, prior treatment history, molecular profile, and patient performance status [5,12]. For isolated locoregional recurrences, salvage surgery, including pelvic exenteration in carefully selected candidates, can yield meaningful improvements in overall survival. In advanced-stage disease, macroscopic cytoreductive resection is pursued when complete tumor clearance is feasible with acceptable morbidity [12]. The first-line systemic regimen for advanced and recurrent EC is carboplatin and paclitaxel (TC), which has been confirmed as non-inferior to the paclitaxel-doxorubicin-cisplatin regimen in the phase III NRG Oncology/GOG0209 trial [13]. Adding the immune checkpoint inhibitor dostarlimab to TC (RUBY trial) has demonstrated statistically significant and clinically meaningful improvements in progression-free and overall survival, particularly in mismatch repair–deficient tumors, establishing a new standard of care for primary advanced and recurrent EC [14].

Acute salpingitis complicating a gynecological malignancy is a rare and diagnostically challenging clinical entity. In postmenopausal women, tubo-ovarian abscess (TOA) accounts for only 6–18% of TOA episodes; in this demographic, an underlying pelvic malignancy must be systematically excluded, as no single clinical parameter, imaging characteristic, or laboratory marker reliably differentiates infectious from neoplastic pelvic pathology [15]. Pelvic procedures such as endometrial biopsy, immunosuppression induced by systemic chemotherapy, disruption of anatomical barriers by tumor extension, and atrophic changes in the aging genital epithelium all predispose elderly patients to ascending pelvic infection. Clinical presentation in this age group is often atypical, characterized by a blunted febrile response, nonspecific pelvic pain, and absence of classical peritoneal signs, resulting in diagnostic delay with potentially serious consequences [16]. Surgical management, including adnexectomy, percutaneous image-guided drainage, or, when indicated, total hysterectomy with bilateral salpingo-oophorectomy, is required when intravenous broad-spectrum antibiotic therapy fails, when abscess rupture is suspected, or when an underlying malignancy cannot be excluded [15]. The association between pelvic inflammatory disease and gynecological cancer risk is increasingly recognized, with large population-based data demonstrating elevated odds of ovarian carcinoma following prior salpingitis episodes, an association attributed to shared inflammatory microenvironmental pathways [17,18].

The co-occurrence of advanced endometrioid endometrial carcinoma (EEC) and acute purulent salpingitis in an elderly patient represents a highly deceptive clinical entity that mimics a primary infectious process, thereby obscuring the classic presentation pattern of gynecological malignancies. The central clinical novelty of this case lies in its challenging diagnostic trap: the presentation in the emergency setting was dominated by severe pelvic pain and markedly elevated inflammatory markers, heavily leaning the initial workup toward pelvic inflammatory disease (PID) or a tubo-ovarian abscess, rather than an underlying endometrial malignancy. This report documents the clinical workup, multi-stage surgical decision-making, and critical management adjustments required when a silent advanced malignancy is masked by acute pelvic sepsis, providing valuable insights for clinicians managing complicated gynecological oncogeriatric cases.

## 2. Case Report

A 78-year-old female patient presented on 3 January 2024 to the General Surgery Department of “Sf. Spiridon” County Emergency Hospital in Iași with complaints of progressive pain in the lower abdomen. Notably, abnormal postmenopausal vaginal bleeding was entirely absent at first presentation. The onset of mild abdominal symptoms was reported 2 years prior, and the symptoms were investigated in other medical institutions, where a diagnosis of a left ovarian cyst was also established. On presentation, the patient was in good overall condition, with stable vital signs. Upon clinical examination, palpation of the abdomen and pelvis revealed a large, tender inferior abdominal mass. Given the dominance of acute lower abdominal pain, markedly elevated systemic inflammatory markers, and an ultrasound showing a massive mixed solid–liquid pelvic mass, the primary diagnostic workup leaned heavily toward a complicated adnexal infectious process or a tubo-ovarian abscess. Consequently, pre-operative advanced staging imaging (computed tomography) and endometrial sampling or biopsy were not pursued at first presentation due to the acute clinical urgency and the initial framing of the lesion as an emergency infectious entity rather than a primary endometrial malignancy. Laboratory investigations obtained at the time of admission are included in Table 1; the other parameters were within normal limits.

To provide a clear overview of the clinical course and streamline the case narrative, the sequence of events, patient status, diagnostic findings, and therapeutic steps are summarized chronologically in Table 2.

The patient was admitted to the General Surgery Department for further investigations to determine a targeted diagnosis and to perform any potentially required surgical intervention. She underwent an abdominal and pelvic ultrasound examination on 4 January 2024, which highlighted an expansive tumor measuring 141/181/168 mm, with an approximate volume of 2270 mL and a heterogeneous structure comprising both solid and liquid components. The tumor appeared well separated from the surrounding structures, and there was no free intraperitoneal fluid. On the same day, bloodwork testing for the oncological marker CA-125 came back positive, with a value of 46.8 UI/ml.

All necessary preoperative examinations were thoroughly conducted, including a cardiology consult on 5 January 2024, which, despite the patient’s history lacking any cardiovascular pathologies, identified several conditions. The patient did not present with dyspnea or chest pain, but cardiac auscultation revealed a systolic murmur corresponding to the mitral valve. Blood pressure was 110/65 mmHg, with a heart rate of 80 bpm. EKG showed negative T waves in V_3_–V_4_, and echocardiography revealed mild hypokinesia of the posterior and inferior left ventricular walls, with an undilated left ventricle (tele-diastolic diameter of 46 mm) and a preserved ejection fraction of 50%. The patient was also diagnosed with grade I mitral regurgitation and grade II-III tricuspid regurgitation. The atrial chambers were normal in volume, and the patient exhibited multiple extrasystoles during the examination, prompting the recommendation of further elective Holter-EKG monitoring.

On 5 January 2024, the patient underwent an emergency open supracervical (subtotal) abdominal hysterectomy with bilateral adnexectomy. Upon comprehensive surgical exploration, intense localized acute inflammatory changes were encountered in the left pelvis, with the left fallopian tube and ovary fused into a multilocular pyogenic mass. Due to severe tissue friability, dense pelvic inflammatory attachments, and the critical clinical necessity to minimize the risk of serious iatrogenic injuries to the adjacent pelvic vessels and duplicated ureteral structures in an emergency general surgery setting, a supracervical hysterectomy was performed as a targeted damage-control approach rather than a standard total abdominal hysterectomy.

**Figure 1:**
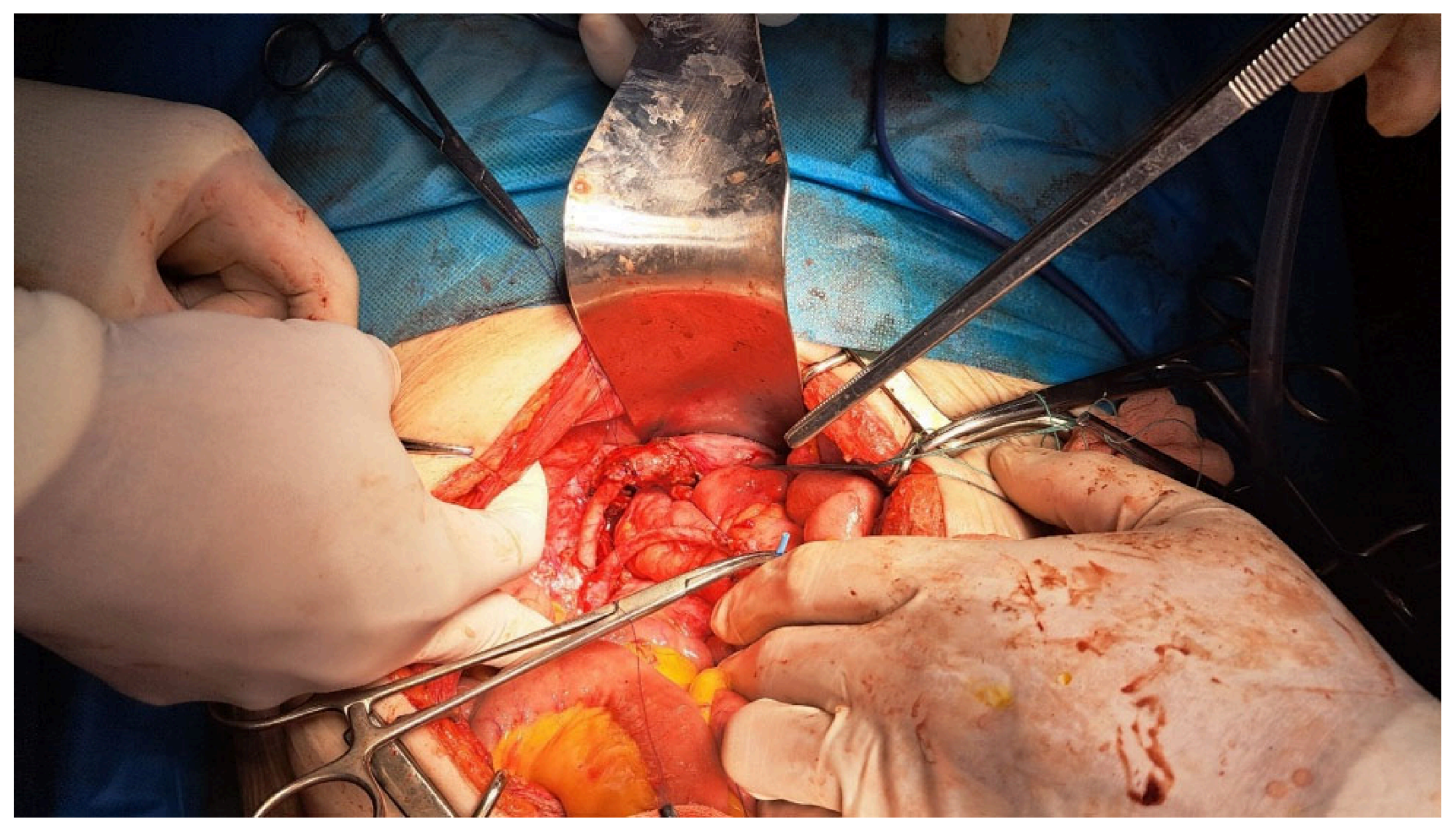
Intraoperative view during the emergency index surgery, demonstrating a voluminous pelvic-abdominal mass arising from the uterine body. The operatory field is characterized by severe tissue friability and intense acute inflammatory changes, with the left adnexa fused into a purulent tubo-ovarian mass, which compelled a damage-control supracervical hysterectomy to prevent major vascular and urological injuries.

The uterus was dissected down to the lower segment, sectioned below the visually apparent boundaries of the mass, and the remaining supracervical uterine stump was closed with heavy absorbable sutures (Figure 1).

The resection specimen was sent for pathological and immunohistochemical examination and consisted of the uterine body, which contained an infiltrative tumor, and the bilateral fallopian tubes and ovaries, with the left tube and ovary fused into a multilocular, cyst-like formation. Abdominal drainage was placed, and sutures were performed on all abdominal wall layers. Following the subsequent histopathological confirmation of an advanced high-risk endometrioid adenocarcinoma with involved surgical margins, immediate completion surgery—including radical cervical removal and pelvic lymph node assessment—along with a post-operative staging computed tomography, were temporarily postponed. This clinical decision was dictated by the patient’s advanced age, severe underlying cardiac status, and the prolonged perioperative recovery period required to completely resolve the concurrent acute purulent salpingitis and parasalpingeal abscesses.

The anatomopathological findings were received on 8 January 2024, confirming the diagnosis of differentiated infiltrative endometrioid endometrial carcinoma, pT3aNxMx, which corresponds to FIGO stage IIIA due to serosal and adnexal involvement. Macroscopic analysis highlighted a 1 cm right ovarian cyst; a fused 6/5.5/3.2 cm mass involving the left ovary and salpinx with areas of necrosis and hemorrhage; and the uterine body with an imprecisely delimited, circumferential tumoral mass (5.5 cm in length and 1.5 cm thickness) in the isthmic area, adjacent to the resection margin, showing an ulcerous-vegetative and infiltrative aspect with areas of necrosis. Optical microscopy revealed a highly infiltrative tumor with glandular or cribriform histological architecture, several areas of squamous differentiation, and areas of necrosis and hemorrhage. The tumor extended outward, penetrating the uterine serosa and demonstrating microscopically involved (positive) surgical resection margins in both the inferior and lateral planes (absent parametria).

There were no signs of vascular permeation or perineural infiltration. The left annexes showed acute purulent salpingitis with parasalpingeal abscesses and secondary ovarian adherences. The immunohistochemistry findings are listed in Table 3.

The patient’s evolution was favorable and was not marked by any severe complications at any point. She resumed gastrointestinal transit of gas and fecal matter, as well as oral intake, from the first postoperative day and developed a mild dyspeptic syndrome. The drainage tube had a steadily decreasing output, allowing its removal on the 5th postoperative day. The patient was discharged on 13 January 2024, with recommendations to follow a hygienic-dietary regimen, change the sterile dressing every two days until the incision is fully healed, and return after 14–21 days for suture removal and surgical evaluation.

Despite initial surgical management and intensive care, the patient presents to the hospital on 25 June 2024, complaining of vaginal bleeding and fatigue. She is admitted to the General Surgery Department I with mildly altered general status. The blood test results are listed in Table 4. Notably, the tumor marker CA-125 was not re-evaluated during this second admission due to local laboratory logistical constraints and the clinical urgency of addressing the active vaginal bleeding. Repeated measurements of RBC and hemoglobin indicate active bleeding, as the hemoglobin gradually decreases from 10 mg/dL upon admission to 9.4 mg/dL 20 h later.

Prior to this admission, the patient underwent a thoraco-abdomino-pelvic CT scan with intravenous contrast on 29 April 2024. This examination provided crucial information about the patient’s particular anatomy, highlighting a sliding hiatal hernia of ~43 mm and an 11 mm hiatal defect; an accessory spleen measuring ~11 mm; a kink of the abdominal aorta; and the presence of two left ureters.

The images also showed several areas of hepatic steatosis and a cyst in liver segment VIII. No free pleural, pericardial, or peritoneal fluid was found, and no enlarged mediastinal or intra-abdominal lymph nodes were present. A mild mesenteric inflammatory reaction was observed. The remaining uterus measured ~47/41/47 mm, had a heterogeneous appearance with a gas bubble in the middle, and showed uneven contrast enhancement, predominant in the periphery. The uterine formation was in close contact with the bladder and the left ureters, generating a mass effect on them and maintaining a fine dissection margin between them.

**Figure 2:**
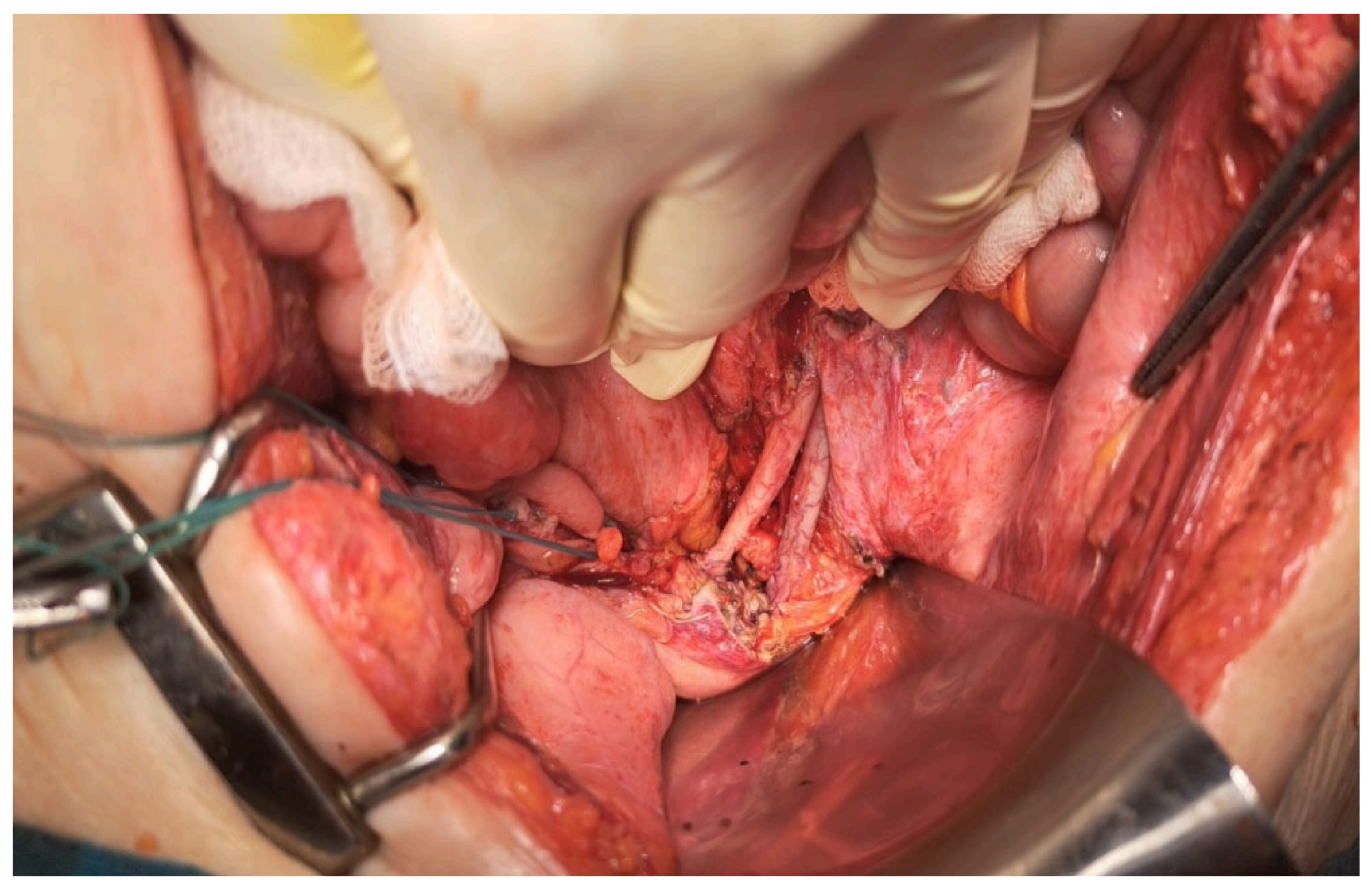
Intraoperative view during the secondary salvage surgery, illustrating the radical excision of the persistent residual cervical stump. The surgical loops isolate the congenital anatomical variant—the duplicated left ureteral system—which was heavily embedded in dense post-inflammatory retroperitoneal fibrosis, requiring meticulous dissection, prophylactic Double-J catheterization, and subsequent successful urological reconstruction.

Considering the patient’s history, her clinical examination on admission, and the contrast computed tomography report, we established the definitive diagnosis of an aggressive progression of locally advanced residual endometrioid endometrial carcinoma. During the five-month interval between the two admissions, the patient was closely monitored clinically, but further advanced imagistic workup was delayed until the acute onset of vaginal bleeding and worsening fatigue prompted this emergency re-evaluation. To address this highly challenging clinical scenario, the case was formally discussed and evaluated by an institutional multidisciplinary tumor board within a specialized surgical oncology unit. Detailed preoperative gynecological, urological, cardiological, and intensive care consultations deemed the patient fit for a secondary salvage surgical intervention aimed at curative cytoreduction and radical excision of the persistent ongoing tumor. Proactively, prophylactic Double-J catheters were cystoscopically inserted to protect ureteral integrity and to precisely guide their surgical identification and repair in the presence of expected severe pelvic fibrosis. On 26 June 2024, the specialized multidisciplinary surgical team performed an exploratory laparotomy and extensive, meticulous adhesiolysis, successfully converting the previous supracervical hysterectomy into a total abdominal hysterectomy through the complete removal of the persistent residual cervix. This was followed by immediate surgical repair of the damaged left duplicated ureters, utilizing direct bladder reimplantation for one ureter and a termino-terminal anastomosis over the indwelling Double-J catheter for the other (Figure 2). The surgery ended with peritoneal lavage for cytologic evaluation, drain placement, and repair of all abdominal wall layers.

Cytology evaluation of the peritoneal lavage fluid was reported on 27 June 2024, showing no signs of tumor cells on the analyzed slides. The anatomopathological examination of the resection specimen described a 5/4.8/5 cm cervix with a 1.7/0.3 cm anterior vaginal resection margin, infiltrated by a compact tumor mass measuring 4.3/4.4/4.9 cm that reaches the distal edge of the cervix, with signs of necrosis, ulceration, and small areas of hemorrhage. The surgical resection margin lay less than 0.1 cm from the tumor tissue, both anteriorly and posteriorly. Optical microscopy highlighted the same tumor type and structure as the previous analysis from 18 January 2024, confirming the diagnosis of recurrent locally advanced differentiated endometrioid endometrial carcinoma, classified as FIGO stage II based on the documented histopathological involvement and infiltration of the cervical stroma. The resection margin was clear of tumor infiltration toward the vaginal stump but in close contact both anteriorly and posteriorly. Contrary to the previous anatomopathological examinations, there were highly suggestive signs of vascular penetration and perineural invasion (LV1Pn1).

The patient had a slow but favorable postoperative course, resuming gastrointestinal transit 2 days post-op and oral intake 3 days post-op. Drain output gradually decreased, and the drain tubes were removed 10 days post-op. The patient was discharged on 9 July 2024, with recommendations to remove sutures (21 days), remove JJ catheters (60 days), wear an abdominal binder for 6 months, follow a hygienic-dietary regimen, and schedule an oncology consult. Following the first surgery in January 2024, adjuvant therapy was discussed by the multidisciplinary tumor board; however, due to the acute infectious complication (purulent salpingitis) and the patient’s immediate recovery needs, systemic treatment was delayed, and the recurrence developed before its clinical initiation. After the salvage surgery in July 2024, the patient was promptly referred to the oncology department and successfully initiated systemic chemotherapy with the Carboplatin-Paclitaxel doublet. Advanced molecular testing for mismatch repair (MMR/MSI) status and POLE mutations was requested but could not be performed due to technical and economic limitations in our unit, which stands as a limitation of this case report. At the most recent follow-up evaluation performed in August 2026, the patient demonstrated a complete clinical and imagistic response with no evidence of further local failure or distant metastasis, maintaining a stable survival status.

## 3. Discussion

This case illustrates a clinically complex presentation combining the rapid and aggressive progression of a persistent residual endometrioid endometrial carcinoma (EEC) with concurrent acute purulent salpingitis in a 78-year-old geriatric patient, a co-occurrence that has not been systematically characterized in the current literature. The clinical significance of this report lies in three critical domains: the diagnostic and surgical challenges posed by the dual pathology during the initial emergency, the atypical and accelerated pattern of residual disease progression rather than a true isolated recurrence, and the extreme technical complexity of the salvage completion surgery in a patient with significant cardiac comorbidities and a duplicated urological anatomy. Framing this case strictly as a primary locoregional recurrence would fail to account for the biological reality of the emergency index surgery; it more accurately represents a rapid ongoing tumor progression dictated by the severe intraoperative inflammatory constraints that necessitated close supracervical resection margins to preserve patient safety. According to the current National Comprehensive Cancer Network (NCCN) and European Society of Gynaecological Oncology (ESGO) guidelines, the standard management for advanced high-risk endometrioid endometrial cancer mandates a total abdominal hysterectomy, bilateral salpingo-oophorectomy, and systematic lymph node staging, followed by prompt adjuvant systemic therapy. However, as demonstrated in this clinical scenario, the presence of a severe, concurrent acute pelvic infection can drastically alter the diagnostic and therapeutic trajectory. At first presentation, the dominant clinical picture was a massive, tender pelvic mass accompanied by marked leukocytosis and neutrophilia.

The underlying acute purulent salpingitis with parasalpingeal abscesses masked the endometrial malignancy, causing a significant pre-operative diagnostic gap. This is highly consistent with the known pattern of atypical and blunted inflammatory presentations in postmenopausal elderly patients, where clinical and laboratory parameters frequently fail to differentiate infectious from neoplastic pelvic pathologies. This diagnostic overlap forced an emergency surgical intervention in a general surgery setting, where dense inflammatory attachments, heavy tissue friability, and the presence of a pyogenic pelvic field compelled the surgical team to perform a supracervical damage-control resection to safeguard the patient’s immediate survival, inadvertently creating a permissive microenvironment for the rapid progression of the residual tumor.

The index surgical procedure, an open supracervical hysterectomy with bilateral adnexectomy, was completed as a life-saving damage-control measure under severe inflammatory constraints. Postoperative histopathological evaluation confirmed microscopically positive surgical margins (R1 resection) in both the inferior and lateral planes, where the tumor directly involved the surgical transection lines. This positive margin status provided the immediate anatomical substrate for the rapid locoregional progression of the residual tumor. The incomplete tumor clearance at the index surgery, forced by the concurrent pelvic sepsis, left viable malignant tissue that drove the subsequent accelerated recurrence interval.

The persistent malignant tissue left by this R1 resection may have facilitated the development of a more aggressive biological phenotype, as evidenced by the subsequent emergence of lymphovascular space invasion and perineural invasion (LV1Pn1), highlighting a potential transition toward an infiltrative and neurotropic biological profile. From a pathophysiological standpoint, this clinical scenario highlights a noteworthy intersection between an aggressive oncogenic drive and a severe, localized pro-inflammatory microenvironment [19]. The tumor’s immunohistochemical profile, featuring prominent estrogen (ER) and progesterone (PR) receptor positivity at 90% alongside a wild-type p53 distribution and negative p16, classically supports a well-differentiated, hormone-dependent Type I model. However, the high Ki67 proliferative index of 50% indicates significant underlying biological replication rates. The existing scientific literature suggests that when such a high proliferative state coexisted within an active, purulent inflammatory macroenvironment—such as that induced by concurrent acute purulent salpingitis and parasalpingeal abscesses—the local up-regulation of pro-inflammatory cytokines, angiogenic factors, and reactive oxygen species can potentially act as a synergistic promoter of tumor recurrence. Although these specific molecular markers were not explicitly examined in our patient, this bio-pathological convergence offers a plausible theoretical explanation for how the tumor overrode its nominally favorable molecular immunophenotype, potentially accelerating tissue infiltration and driving the rapid five-month locoregional progression observed in this geriatric patient.

The second procedure was substantially more complex than the first operation. The CT evaluation obtained prior to the second admission identified a 47 mm heterogeneous residual uterine formation in direct contact with the bladder and the left ureters, compounded by a congenital duplication of the left ureter, an anatomical variant with direct intraoperative consequences. Preoperative Double-J catheter insertion was a mandatory safety measure that both facilitated ureteral identification during extensive adhesiolysis and enabled immediate repair when iatrogenic injury occurred. The need to perform bladder reimplantation for one ureter and termino-terminal anastomosis over the JJ catheter for the other, concurrently with the completion total hysterectomy, illustrates the level of technical versatility required when salvage surgery is undertaken in a setting of prior incomplete resection, locoregional adhesive disease, and complex anatomy. The cardiac comorbidities discovered at preoperative assessment, including left ventricular hypokinesia, preserved but borderline ejection fraction of 50%, and valvular regurgitation, further elevated the perioperative risk and required multidisciplinary coordination across gynecological oncology, urology, cardiology, and intensive care.

The second anatomopathological analysis demonstrated disease progression beyond the initial staging, with confirmed vascular and perineural invasion (LV1Pn1) absent in the index specimen, indicating biological aggressiveness of the recurrent tumor. The resection margin, though free of tumoral infiltration at the vaginal stump, was within 0.1 cm anteriorly and posteriorly, conferring a high risk of further local failure. The negative peritoneal cytology was the sole favorable prognostic indicator from the operative assessment. Per current ESGO-ESTRO-ESP guidelines, the subsequent oncological management of this patient mandates systemic therapy, with the carboplatin-paclitaxel doublet constituting the established first-line regimen for advanced and recurrent EC, with the addition of immune checkpoint inhibition to be considered according to mismatch repair status [12,13,14].

## 4. Conclusions

This case highlights a highly deceptive clinical scenario where an advanced gynecological malignancy was completely masked by the features of an acute, concurrent pelvic infection. The key clinical lesson is that in elderly postmenopausal women, locally advanced endometrial cancer can present with atypical features—such as severe lower abdominal pain and systemic inflammatory response—mimicking pelvic inflammatory disease, acute salpingitis, or a pelvic abscess, while entirely lacking the classic warning sign of abnormal vaginal bleeding. Such an overlapping infectious presentation can obscure the standard diagnostic trajectory and obscure the underlying malignancy, driving emergency, non-radical surgical interventions. Clinicians must maintain a high index of suspicion for hidden neoplastic processes when managing severe pelvic sepsis in the geriatric demographic. When incomplete index resections are forced by severe pelvic sepsis, a planned, secondary multidisciplinary strategy integrating oncology, gynecology, and urology remains vital to safely execute salvage completion surgery and achieve complete oncological clearance.

## Figures and Tables

**Table 1:** Laboratory parameters on first admission (3 January 2024).

Laboratory Investigations	Value	Normal Values
Blood Glucose	125 mg/dL	<110 mg/dL
Urea	53 mg/dL	<23 mg/dL
Alkaline phosphatase (ALP)	149 U/L	30–130 U/L
Total Proteins	8.18 g/dL	6–8 g/dL
White blood cells	10,980/μL	4000–10,000/μL
Red blood cells	4,320,000/μL	3,100,000–5,200,000/μL
Hemoglobin	11.8 g/dL	12–15.5 g/dL
Neutrophils (%)	82.6%	45–80%
Lymphocytes (%)	7.8%	20–45%

**Table 2:** Chronological timeline of clinical presentations, diagnostic findings, and multi-stage therapeutic interventions.

Timeline	Clinical Presentation & Patient Status	Diagnostic Investigations	Therapeutic Interventions	Key Surgical & Pathological Findings
Initial Presentation (January 2024)	Acute progressive lower abdominal pain Tender palpable inferior abdominal mass Absence of postmenopausal vaginal bleeding ECOG Performance Status: 1 ASA Physical Status: II	Leukocytosis: 10,900/µL Neutrophils: 82.6% CA-125: 46.8 U/mL Pelvic US: Mixed solid-liquid mass (141/181/168 mm, ~2270 mL)	Emergency open supracervical (subtotal) abdominal hysterectomy with bilateral adnexectomy	Differentiated infiltrative endometrioid endometrial carcinoma, pT3aNxMx (FIGO stage IIIA) Complication: Fused left adnexa with acute purulent salpingitis and parasalpingeal abscesses Margins: Microscopically involved/positive inferior and lateral margins
Interim Outpatient Monitoring (April 2024)	Asymptomatic clinical status Postoperative recovery from pelvic sepsis completed ECOG Performance Status: 1	Thoraco-abdomino-pelvic contrast CT (29 April, 2024): Heterogeneous residual uterine mass (~47/41/47 mm) invading the residual cerva, in close contact with the bladder Anatomical variant: Congenital left duplicated ureters	Institutional Multidisciplinary Tumor Board (MDT) evaluation Scheduling of secondary salvage radical surgery	Confirmation of rapid locoregional progression of residual disease Comprehensive surgical-urological planning for complex anatomy
Emergency Re-admission (June 2024)	Acute postmenopausal vaginal bleeding Progressive fatigue ECOG Performance Status: 2 ASA Physical Status: III (due to active hemorrhage & cardiac status)	Anemia: Hemoglobin dropped from 10 g/dL to 9.4 g/dL within 20 h Platelets: 416,000/µL Peritoneal Lavage Cytology: Negative for malignant cells	Prophylactic cystoscopic Double-J catheter insertion Salvage completion surgery: Exploratory laparotomy, total hysterectomy (cervical stump removal), and left duplicated ureteral repair (bladder reimplantation and end-to-end anastomosis)	Pathology: Recurrent/progressive locally advanced EEC involving the cervical stroma (FIGO Stage II) Upgraded biology: Microscopic presence of lymphovascular space invasion and perineural invasion (LV1Pn1) Margins: Close (<0.1 cm)
Adjuvant Treatment & Long-term Follow-up (July 2024–August 2026)	Slow but favorable postoperative course Complete clinical and imagistic response at 26 months post-salvage surgery ECOG Performance Status: 0	Regular clinical oncological assessments and follow-up imaging	Referral to Medical Oncology Department Initiation of systemic doublet chemotherapy (Carboplatin + Paclitaxel protocol)	No evidence of further local failure or distant metastasis Patient maintains a stable, disease-free survival status

**Table 3:** IHC findings (received on the 18 January 2024).

Investigated Parameters & Markers	Values & Distribution
Ck7	positive in 70% of tumoral cells
ER	90% positive, intensity level 2–3
PR	90% positive, intensity level 2–3
CEA	positive
CD10	positive in tumoral stroma
Ki67	50%
P53	positive in isolated cells, wild-type
Vimentin	positive in isolated cells
P16	negative, isolated nuclear (5%), isolated cytoplasmatic (30%)
CK20, GATA3, TTF1	negative

**Table 4:** Laboratory parameters on second admission (25 June 2024).

Laboratory Investigations	Value	Normal Values
Blood Glucose	97 mg/dL	<110 mg/dL
Urea	43 mg/dL	<23 mg/dL
Alkaline phosphatase (ALP)	118 U/L	30–130 U/L
Pancreatic amylase	104 U/L	30–110 U/L
C Reactive Proteine	1.03 mg/dL	<0.5 mg/dL
White blood cells	7830/μL	4000–10,000/μL
Red blood cells	3,580,000/μL	3,100,000–5,200,000/μL
Hemoglobin	10 g/dL	12–15.5 g/dL
Neutrophils (%)	70%	45–80%
Lymphocytes (%)	19.7%	20–45%
Platelet count	416,000/μL	150,000–350,000/μL

## Data Availability

The original contributions presented in this study are included in the article. Further inquiries can be directed to the corresponding author.
